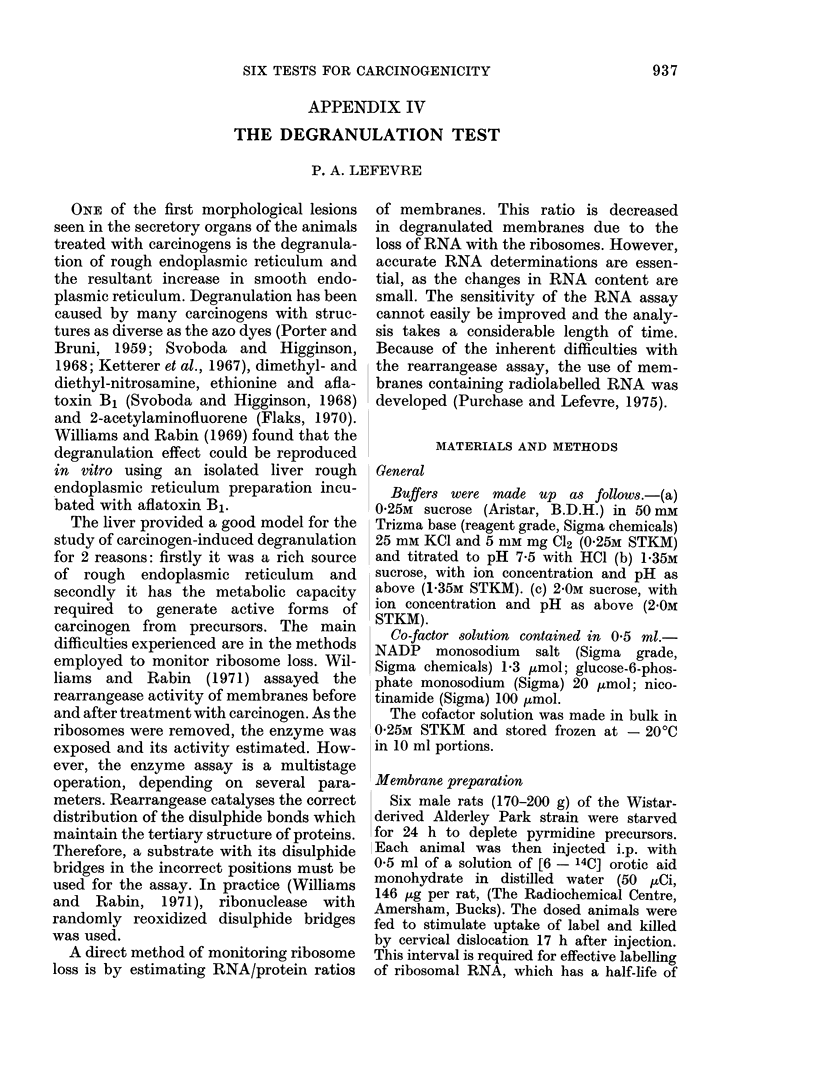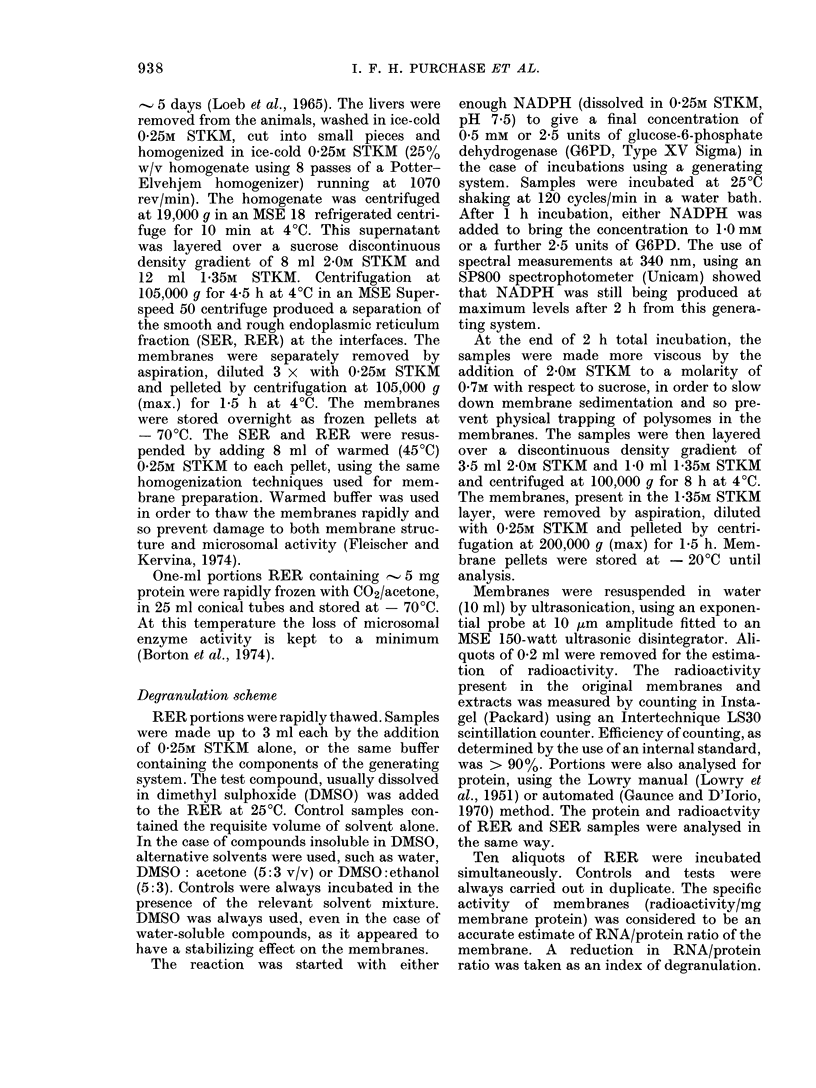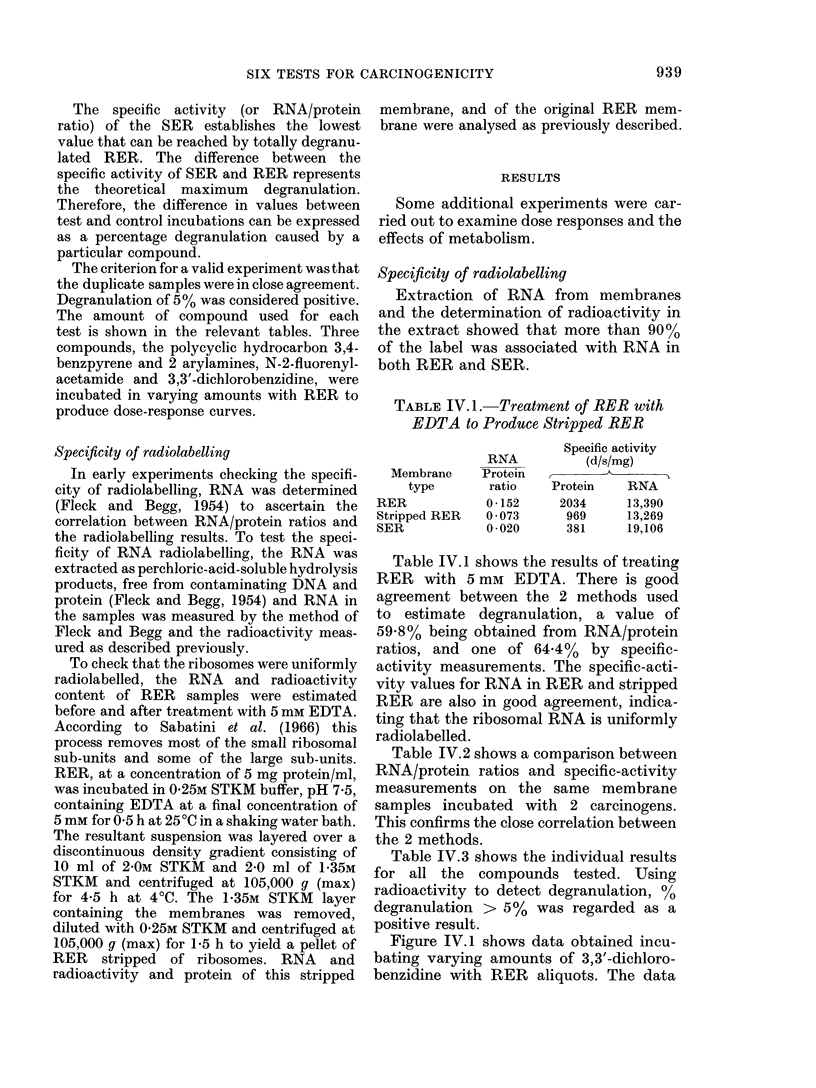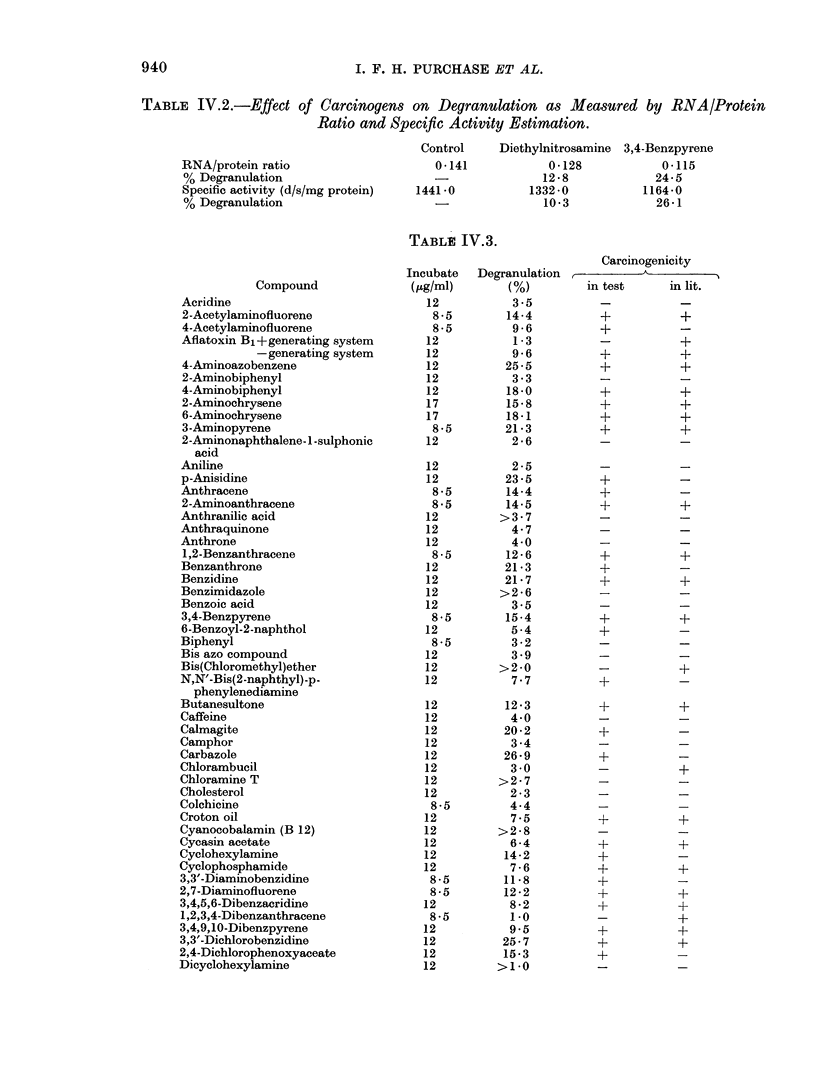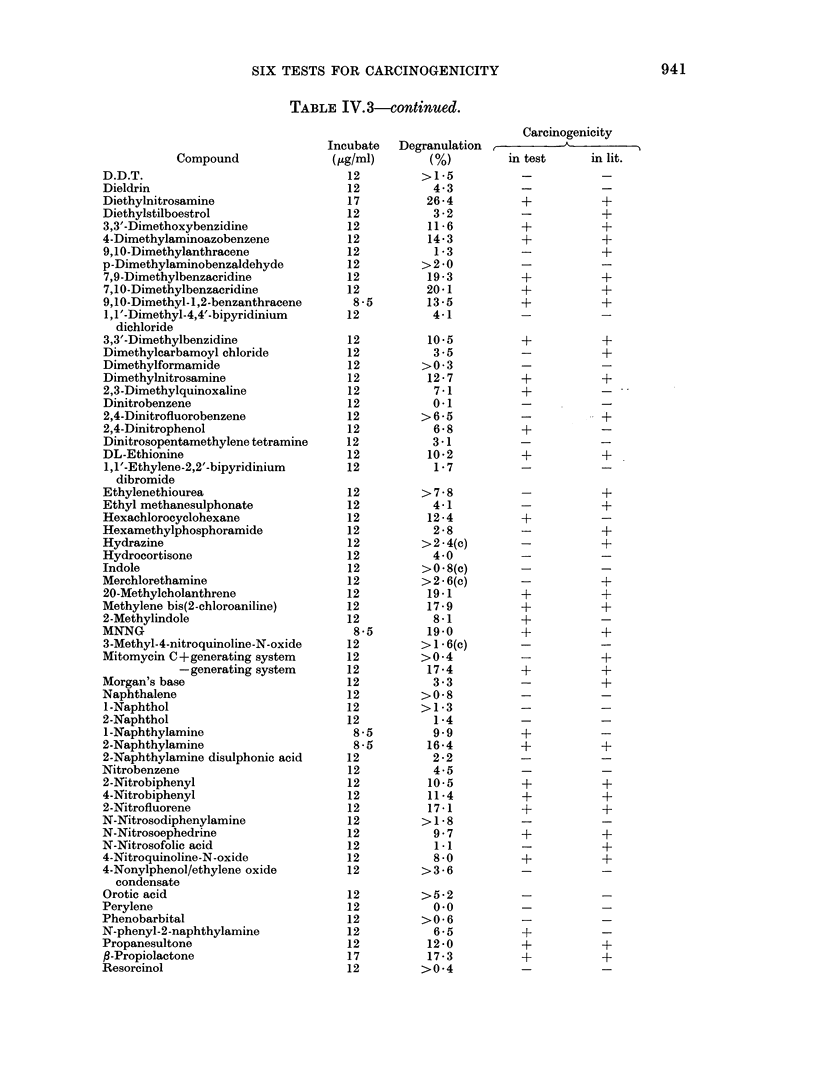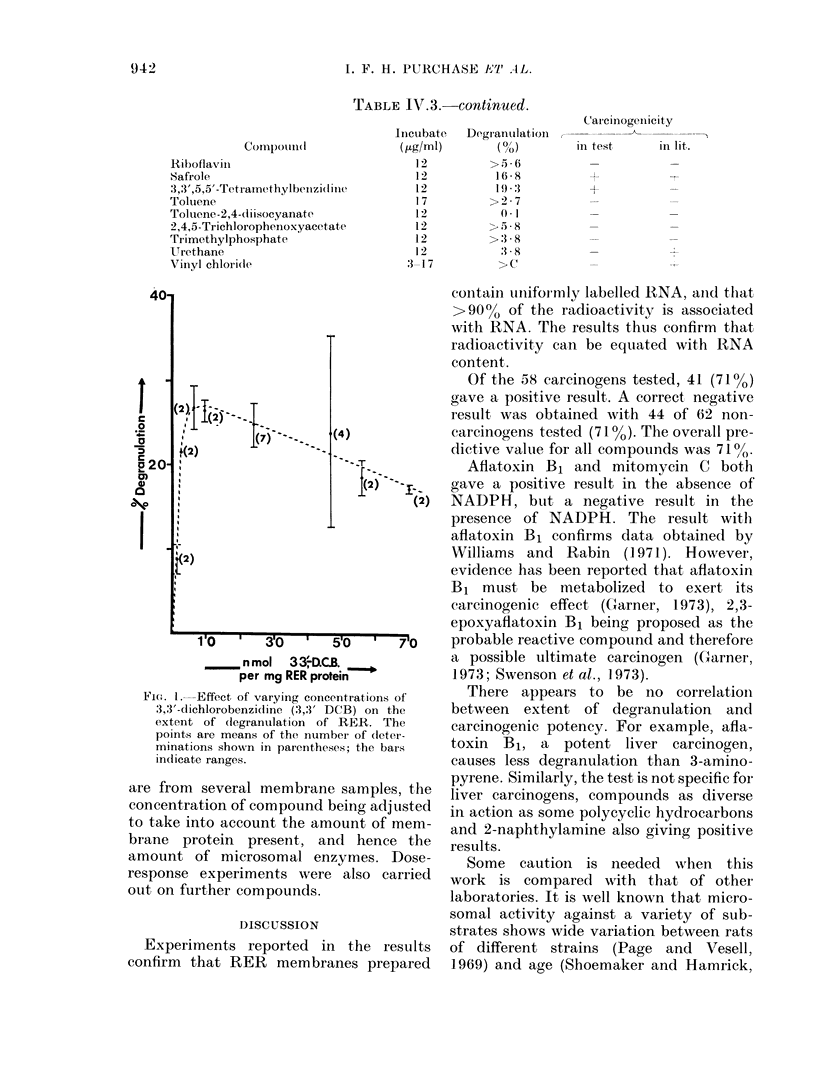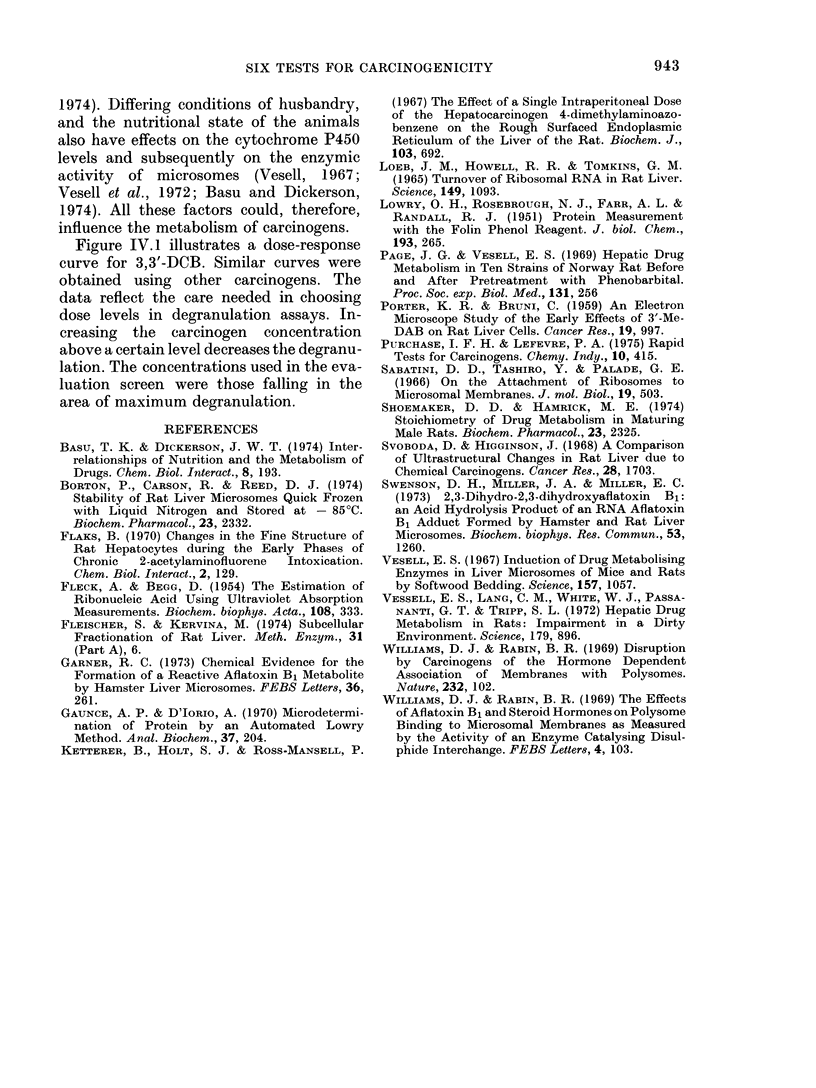# The degranulation test. Six tests for carcinogenicity.

**DOI:** 10.1038/bjc.1978.136

**Published:** 1978-06

**Authors:** P. A. Lefevre


### APPENDIX IV

### THE DEGRANULATION TEST

P. A. LEFEVRE

ONE of the first morphological lesions seen in the secretory organs of the animals treated with carcinogens is the degranulation of rough endoplasmic reticulum and the resultant increase in smooth endoplasmic reticulum. Degranulation has been caused by many carcinogens with structures as diverse as the azo dyes (Porter and Bruni, 1959; Svoboda and Higginson, 1968; Ketterer *et al.*, 1967), dimethyl- and diethyl-nitrosamine, ethionine and aflatoxin $B_1$ (Svoboda and Higginson, 1968) and 2-acetylaminofluorene (Flaks, 1970). Williams and Rabin (1969) found that the degranulation effect could be reproduced *in vitro* using an isolated liver rough endoplasmic reticulum preparation incubated with aflatoxin $B_1$.

The liver provided a good model for the study of carcinogen-induced degranulation for 2 reasons: firstly it was a rich source of rough endoplasmic reticulum and secondly it has the metabolic capacity required to generate active forms of carcinogen from precursors. The main difficulties experienced are in the methods employed to monitor ribosome loss. Williams and Rabin (1971) assayed the rearrangease activity of membranes before and after treatment with carcinogen. As the ribosomes were removed, the enzyme was exposed and its activity estimated. However, the enzyme assay is a multistage operation, depending on several parameters. Rearrangease catalyses the correct distribution of the disulphide bonds which maintain the tertiary structure of proteins. Therefore, a substrate with its disulphide bridges in the incorrect positions must be used for the assay. In practice (Williams and Rabin, 1971), ribonuclease with randomly reoxidized disulphide bridges was used.

A direct method of monitoring ribosome loss is by estimating RNA/protein ratios of membranes. This ratio is decreased in degranulated membranes due to the loss of RNA with the ribosomes. However, accurate RNA determinations are essential, as the changes in RNA content are small. The sensitivity of the RNA assay cannot easily be improved and the analysis takes a considerable length of time. Because of the inherent difficulties with the rearrangease assay, the use of membranes containing radiolabelled RNA was developed (Purchase and Lefevre, 1975).

### MATERIALS AND METHODS

### General

*Buffers were made up as follows.*—(a) 0·25M sucrose (Aristar, B.D.H.) in 50 mM Trizma base (reagent grade, Sigma chemicals) 25 mM KCl and 5 mM mg $Cl_2$ (0·25M STKM) and titrated to pH 7·5 with HCl (b) 1·35M sucrose, with ion concentration and pH as above (1·35M STKM). (c) 2·0M sucrose, with ion concentration and pH as above (2·0M STKM).

*Co-factor solution contained in 0·5 ml.*—NADP monosodium salt (Sigma grade, Sigma chemicals) 1·3 μmol; glucose-6-phosphate monosodium (Sigma) 20 μmol; nicotinamide (Sigma) 100 μmol.

The cofactor solution was made in bulk in 0·25M STKM and stored frozen at − 20°C in 10 ml portions.

### Membrane preparation

Six male rats (170–200 g) of the Wistar-derived Alderley Park strain were starved for 24 h to deplete pyrmidine precursors. Each animal was then injected i.p. with 0·5 ml of a solution of [6 − $^{14}C$] orotic aid monohydrate in distilled water (50 μCi, 146 μg per rat, (The Radiochemical Centre, Amersham, Bucks). The dosed animals were fed to stimulate uptake of label and killed by cervical dislocation 17 h after injection. This interval is required for effective labelling of ribosomal RNA, which has a half-life of

~ 5 days (Loeb *et al.*, 1965). The livers were removed from the animals, washed in ice-cold 0·25M STKM, cut into small pieces and homogenized in ice-cold 0·25M STKM (25% w/v homogenate using 8 passes of a Potter-Elvehjem homogenizer) running at 1070 rev/min). The homogenate was centrifuged at 19,000 *g* in an MSE 18 refrigerated centrifuge for 10 min at 4°C. This supernatant was layered over a sucrose discontinuous density gradient of 8 ml 2·0M STKM and 12 ml 1·35M STKM. Centrifugation at 105,000 *g* for 4·5 h at 4°C in an MSE Superspeed 50 centrifuge produced a separation of the smooth and rough endoplasmic reticulum fraction (SER, RER) at the interfaces. The membranes were separately removed by aspiration, diluted 3 × with 0·25M STKM and pelleted by centrifugation at 105,000 *g* (max.) for 1·5 h at 4°C. The membranes were stored overnight as frozen pellets at − 70°C. The SER and RER were resuspended by adding 8 ml of warmed (45°C) 0·25M STKM to each pellet, using the same homogenization techniques used for membrane preparation. Warmed buffer was used in order to thaw the membranes rapidly and so prevent damage to both membrane structure and microsomal activity (Fleischer and Kervina, 1974).

One-ml portions RER containing ~ 5 mg protein were rapidly frozen with $CO_2$/acetone, in 25 ml conical tubes and stored at − 70°C. At this temperature the loss of microsomal enzyme activity is kept to a minimum (Borton *et al.*, 1974).

*Degranulation scheme*

RER portions were rapidly thawed. Samples were made up to 3 ml each by the addition of 0·25M STKM alone, or the same buffer containing the components of the generating system. The test compound, usually dissolved in dimethyl sulphoxide (DMSO) was added to the RER at 25°C. Control samples contained the requisite volume of solvent alone. In the case of compounds insoluble in DMSO, alternative solvents were used, such as water, DMSO : acetone (5:3 v/v) or DMSO:ethanol (5:3). Controls were always incubated in the presence of the relevant solvent mixture. DMSO was always used, even in the case of water-soluble compounds, as it appeared to have a stabilizing effect on the membranes.

The reaction was started with either enough NADPH (dissolved in 0·25M STKM, pH 7·5) to give a final concentration of 0·5 mM or 2·5 units of glucose-6-phosphate dehydrogenase (G6PD, Type XV Sigma) in the case of incubations using a generating system. Samples were incubated at 25°C shaking at 120 cycles/min in a water bath. After 1 h incubation, either NADPH was added to bring the concentration to 1·0 mM or a further 2·5 units of G6PD. The use of spectral measurements at 340 nm, using an SP800 spectrophotometer (Unicam) showed that NADPH was still being produced at maximum levels after 2 h from this generating system.

At the end of 2 h total incubation, the samples were made more viscous by the addition of 2·0M STKM to a molarity of 0·7M with respect to sucrose, in order to slow down membrane sedimentation and so prevent physical trapping of polysomes in the membranes. The samples were then layered over a discontinuous density gradient of 3·5 ml 2·0M STKM and 1·0 ml 1·35M STKM and centrifuged at 100,000 *g* for 8 h at 4°C. The membranes, present in the 1·35M STKM layer, were removed by aspiration, diluted with 0·25M STKM and pelleted by centrifugation at 200,000 *g* (max) for 1·5 h. Membrane pellets were stored at − 20°C until analysis.

Membranes were resuspended in water (10 ml) by ultrasonication, using an exponential probe at 10 μm amplitude fitted to an MSE 150-watt ultrasonic disintegrator. Aliquots of 0·2 ml were removed for the estimation of radioactivity. The radioactivity present in the original membranes and extracts was measured by counting in Instagel (Packard) using an Intertechnique LS30 scintillation counter. Efficiency of counting, as determined by the use of an internal standard, was > 90%. Portions were also analysed for protein, using the Lowry manual (Lowry *et al.*, 1951) or automated (Gaunce and D'Iorio, 1970) method. The protein and radioactvity of RER and SER samples were analysed in the same way.

Ten aliquots of RER were incubated simultaneously. Controls and tests were always carried out in duplicate. The specific activity of membranes (radioactivity/mg membrane protein) was considered to be an accurate estimate of RNA/protein ratio of the membrane. A reduction in RNA/protein ratio was taken as an index of degranulation.

The specific activity (or RNA/protein ratio) of the SER establishes the lowest value that can be reached by totally degranulated RER. The difference between the specific activity of SER and RER represents the theoretical maximum degranulation. Therefore, the difference in values between test and control incubations can be expressed as a percentage degranulation caused by a particular compound.

The criterion for a valid experiment was that the duplicate samples were in close agreement. Degranulation of 5% was considered positive. The amount of compound used for each test is shown in the relevant tables. Three compounds, the polycyclic hydrocarbon 3,4-benzpyrene and 2 arylamines, N-2-fluorenylacetamide and 3,3′-dichlorobenzidine, were incubated in varying amounts with RER to produce dose-response curves.

*Specificity of radiolabelling*

In early experiments checking the specificity of radiolabelling, RNA was determined (Fleck and Begg, 1954) to ascertain the correlation between RNA/protein ratios and the radiolabelling results. To test the specificity of RNA radiolabelling, the RNA was extracted as perchloric-acid-soluble hydrolysis products, free from contaminating DNA and protein (Fleck and Begg, 1954) and RNA in the samples was measured by the method of Fleck and Begg and the radioactivity measured as described previously.

To check that the ribosomes were uniformly radiolabelled, the RNA and radioactivity content of RER samples were estimated before and after treatment with 5 mM EDTA. According to Sabatini *et al.* (1966) this process removes most of the small ribosomal sub-units and some of the large sub-units. RER, at a concentration of 5 mg protein/ml, was incubated in 0·25M STKM buffer, pH 7·5, containing EDTA at a final concentration of 5 mM for 0·5 h at 25 °C in a shaking water bath. The resultant suspension was layered over a discontinuous density gradient consisting of 10 ml of 2·0M STKM and 2·0 ml of 1·35M STKM and centrifuged at 105,000 *g* (max) for 4·5 h at 4°C. The 1·35M STKM layer containing the membranes was removed, diluted with 0·25M STKM and centrifuged at 105,000 *g* (max) for 1·5 h to yield a pellet of RER stripped of ribosomes. RNA and radioactivity and protein of this stripped membrane, and of the original RER membrane were analysed as previously described.

RESULTS

Some additional experiments were carried out to examine dose responses and the effects of metabolism.

*Specificity of radiolabelling*

Extraction of RNA from membranes and the determination of radioactivity in the extract showed that more than 90% of the label was associated with RNA in both RER and SER.

TABLE IV.1.—*Treatment of RER with EDTA to Produce Stripped RER*

| Membrane type | RNA/Protein ratio | Specific activity (d/s/mg) | |
|---|---|---|---|
| | | Protein | RNA |
| RER | 0·152 | 2034 | 13,390 |
| Stripped RER | 0·073 | 969 | 13,269 |
| SER | 0·020 | 381 | 19,106 |

Table IV.1 shows the results of treating RER with 5 mM EDTA. There is good agreement between the 2 methods used to estimate degranulation, a value of 59·8% being obtained from RNA/protein ratios, and one of 64·4% by specific-activity measurements. The specific-activity values for RNA in RER and stripped RER are also in good agreement, indicating that the ribosomal RNA is uniformly radiolabelled.

Table IV.2 shows a comparison between RNA/protein ratios and specific-activity measurements on the same membrane samples incubated with 2 carcinogens. This confirms the close correlation between the 2 methods.

Table IV.3 shows the individual results for all the compounds tested. Using radioactivity to detect degranulation, % degranulation > 5% was regarded as a positive result.

Figure IV.1 shows data obtained incubating varying amounts of 3,3′-dichlorobenzidine with RER aliquots. The data

TABLE IV.2.—*Effect of Carcinogens on Degranulation as Measured by RNA/Protein Ratio and Specific Activity Estimation.*

| | Control | Diethylnitrosamine | 3,4-Benzpyrene |
|---|---|---|---|
| RNA/protein ratio | 0·141 | 0·128 | 0·115 |
| % Degranulation | — | 12·8 | 24·5 |
| Specific activity (d/s/mg protein) | 1441·0 | 1332·0 | 1164·0 |
| % Degranulation | — | 10·3 | 26·1 |

TABLE IV.3.

| Compound | Incubate (μg/ml) | Degranulation (%) | Carcinogenicity in test | Carcinogenicity in lit. |
|---|---|---|---|---|
| Acridine | 12 | 3·5 | — | — |
| 2-Acetylaminofluorene | 8·5 | 14·4 | + | + |
| 4-Acetylaminofluorene | 8·5 | 9·6 | + | — |
| Aflatoxin $B_1$ + generating system | 12 | 1·3 | — | + |
| — generating system | 12 | 9·6 | + | + |
| 4-Aminoazobenzene | 12 | 25·5 | + | + |
| 2-Aminobiphenyl | 12 | 3·3 | — | — |
| 4-Aminobiphenyl | 12 | 18·0 | + | + |
| 2-Aminochrysene | 17 | 15·8 | + | + |
| 6-Aminochrysene | 17 | 18·1 | + | + |
| 3-Aminopyrene | 8·5 | 21·3 | + | + |
| 2-Aminonaphthalene-1-sulphonic acid | 12 | 2·6 | — | — |
| Aniline | 12 | 2·5 | — | — |
| p-Anisidine | 12 | 23·5 | + | — |
| Anthracene | 8·5 | 14·4 | + | — |
| 2-Aminoanthracene | 8·5 | 14·5 | + | + |
| Anthranilic acid | 12 | >3·7 | — | — |
| Anthraquinone | 12 | 4·7 | — | — |
| Anthrone | 12 | 4·0 | — | — |
| 1,2-Benzanthracene | 8·5 | 12·6 | + | + |
| Benzanthrone | 12 | 21·3 | + | — |
| Benzidine | 12 | 21·7 | + | + |
| Benzimidazole | 12 | >2·6 | — | — |
| Benzoic acid | 12 | 3·5 | — | — |
| 3,4-Benzpyrene | 8·5 | 15·4 | + | + |
| 6-Benzoyl-2-naphthol | 12 | 5·4 | + | — |
| Biphenyl | 8·5 | 3·2 | — | — |
| Bis azo compound | 12 | 3·9 | — | — |
| Bis(Chloromethyl)ether | 12 | >2·0 | — | + |
| N,N′-Bis(2-naphthyl)-p-phenylenediamine | 12 | 7·7 | + | — |
| Butanesultone | 12 | 12·3 | + | + |
| Caffeine | 12 | 4·0 | — | — |
| Calmagite | 12 | 20·2 | + | — |
| Camphor | 12 | 3·4 | — | — |
| Carbazole | 12 | 26·9 | + | — |
| Chlorambucil | 12 | 3·0 | — | + |
| Chloramine T | 12 | >2·7 | — | — |
| Cholesterol | 12 | 2·3 | — | — |
| Colchicine | 8·5 | 4·4 | — | — |
| Croton oil | 12 | 7·5 | + | + |
| Cyanocobalamin (B 12) | 12 | >2·8 | — | — |
| Cycasin acetate | 12 | 6·4 | + | + |
| Cyclohexylamine | 12 | 14·2 | + | — |
| Cyclophosphamide | 12 | 7·6 | + | + |
| 3,3′-Diaminobenzidine | 8·5 | 11·8 | + | — |
| 2,7-Diaminofluorene | 8·5 | 12·2 | + | + |
| 3,4,5,6-Dibenzacridine | 12 | 8·2 | + | + |
| 1,2,3,4-Dibenzanthracene | 8·5 | 1·0 | — | + |
| 3,4,9,10-Dibenzpyrene | 12 | 9·5 | + | + |
| 3,3′-Dichlorobenzidine | 12 | 25·7 | + | + |
| 2,4-Dichlorophenoxyaceate | 12 | 15·3 | + | — |
| Dicyclohexylamine | 12 | >1·0 | — | — |

TABLE IV.3—*continued.*

| Compound | Incubate ($\mu$g/ml) | Degranulation (%) | Carcinogenicity in test | Carcinogenicity in lit. |
|---|---|---|---|---|
| D.D.T. | 12 | >1·5 | − | − |
| Dieldrin | 12 | 4·3 | − | − |
| Diethylnitrosamine | 17 | 26·4 | + | + |
| Diethylstilboestrol | 12 | 3·2 | − | + |
| 3,3′-Dimethoxybenzidine | 12 | 11·6 | + | + |
| 4-Dimethylaminoazobenzene | 12 | 14·3 | + | + |
| 9,10-Dimethylanthracene | 12 | 1·3 | − | + |
| p-Dimethylaminobenzaldehyde | 12 | >2·0 | − | − |
| 7,9-Dimethylbenzacridine | 12 | 19·3 | + | + |
| 7,10-Dimethylbenzacridine | 12 | 20·1 | + | + |
| 9,10-Dimethyl-1,2-benzanthracene | 8·5 | 13·5 | + | + |
| 1,1′-Dimethyl-4,4′-bipyridinium dichloride | 12 | 4·1 | − | − |
| 3,3′-Dimethylbenzidine | 12 | 10·5 | + | + |
| Dimethylcarbamoyl chloride | 12 | 3·5 | − | + |
| Dimethylformamide | 12 | >0·3 | − | − |
| Dimethylnitrosamine | 12 | 12·7 | + | + |
| 2,3-Dimethylquinoxaline | 12 | 7·1 | + | − |
| Dinitrobenzene | 12 | 0·1 | − | − |
| 2,4-Dinitrofluorobenzene | 12 | >6·5 | − | + |
| 2,4-Dinitrophenol | 12 | 6·8 | + | − |
| Dinitrosopentamethylene tetramine | 12 | 3·1 | − | − |
| DL-Ethionine | 12 | 10·2 | + | + |
| 1,1′-Ethylene-2,2′-bipyridinium dibromide | 12 | 1·7 | − | − |
| Ethylenethiourea | 12 | >7·8 | − | + |
| Ethyl methanesulphonate | 12 | 4·1 | − | + |
| Hexachlorocyclohexane | 12 | 12·4 | + | − |
| Hexamethylphosphoramide | 12 | 2·8 | − | + |
| Hydrazine | 12 | >2·4(c) | − | + |
| Hydrocortisone | 12 | 4·0 | − | − |
| Indole | 12 | >0·8(c) | − | − |
| Merchlorethamine | 12 | >2·6(c) | − | + |
| 20-Methylcholanthrene | 12 | 19·1 | + | + |
| Methylene bis(2-chloroaniline) | 12 | 17·9 | + | + |
| 2-Methylindole | 12 | 8·1 | + | − |
| MNNG | 8·5 | 19·0 | + | + |
| 3-Methyl-4-nitroquinoline-N-oxide | 12 | >1·6(c) | − | − |
| Mitomycin C + generating system | 12 | >0·4 | − | + |
| − generating system | 12 | 17·4 | + | + |
| Morgan's base | 12 | 3·3 | − | + |
| Naphthalene | 12 | >0·8 | − | − |
| 1-Naphthol | 12 | >1·3 | − | − |
| 2-Naphthol | 12 | 1·4 | − | − |
| 1-Naphthylamine | 8·5 | 9·9 | + | − |
| 2-Naphthylamine | 8·5 | 16·4 | + | + |
| 2-Naphthylamine disulphonic acid | 12 | 2·2 | − | − |
| Nitrobenzene | 12 | 4·5 | − | − |
| 2-Nitrobiphenyl | 12 | 10·5 | + | + |
| 4-Nitrobiphenyl | 12 | 11·4 | + | + |
| 2-Nitrofluorene | 12 | 17·1 | + | + |
| N-Nitrosodiphenylamine | 12 | >1·8 | − | − |
| N-Nitrosoephedrine | 12 | 9·7 | + | + |
| N-Nitrosofolic acid | 12 | 1·1 | − | + |
| 4-Nitroquinoline-N-oxide | 12 | 8·0 | + | + |
| 4-Nonylphenol/ethylene oxide condensate | 12 | >3·6 | − | − |
| Orotic acid | 12 | >5·2 | − | − |
| Perylene | 12 | 0·0 | − | − |
| Phenobarbital | 12 | >0·6 | − | − |
| N-phenyl-2-naphthylamine | 12 | 6·5 | + | − |
| Propanesultone | 12 | 12·0 | + | + |
| $\beta$-Propiolactone | 17 | 17·3 | + | + |
| Resorcinol | 12 | >0·4 | − | − |

TABLE IV.3.—*continued.*

| Compound | Incubate ($\mu$g/ml) | Degranulation (%) | Carcinogenicity in test | Carcinogenicity in lit. |
|---|---|---|---|---|
| Riboflavin | 12 | >5·6 | − | − |
| Safrole | 12 | 16·8 | + | + |
| 3,3′,5,5′-Tetramethylbenzidine | 12 | 19·3 | + | − |
| Toluene | 17 | >2·7 | − | − |
| Toluene-2,4-diisocyanate | 12 | 0·1 | − | − |
| 2,4,5-Trichlorophenoxyacetate | 12 | >5·8 | − | − |
| Trimethylphosphate | 12 | >3·8 | − | − |
| Urethane | 12 | 3·8 | − | + |
| Vinyl chloride | 3–17 | >C | − | + |

FIG. 1.—Effect of varying concentrations of 3,3′-dichlorobenzidine (3,3′ DCB) on the extent of degranulation of RER. The points are means of the number of determinations shown in parentheses; the bars indicate ranges.

are from several membrane samples, the concentration of compound being adjusted to take into account the amount of membrane protein present, and hence the amount of microsomal enzymes. Dose-response experiments were also carried out on further compounds.

### DISCUSSION

Experiments reported in the results confirm that RER membranes prepared contain uniformly labelled RNA, and that >90% of the radioactivity is associated with RNA. The results thus confirm that radioactivity can be equated with RNA content.

Of the 58 carcinogens tested, 41 (71%) gave a positive result. A correct negative result was obtained with 44 of 62 non-carcinogens tested (71%). The overall predictive value for all compounds was 71%.

Aflatoxin $B_1$ and mitomycin C both gave a positive result in the absence of NADPH, but a negative result in the presence of NADPH. The result with aflatoxin $B_1$ confirms data obtained by Williams and Rabin (1971). However, evidence has been reported that aflatoxin $B_1$ must be metabolized to exert its carcinogenic effect (Garner, 1973), 2,3-epoxyaflatoxin $B_1$ being proposed as the probable reactive compound and therefore a possible ultimate carcinogen (Garner, 1973; Swenson *et al.*, 1973).

There appears to be no correlation between extent of degranulation and carcinogenic potency. For example, aflatoxin $B_1$, a potent liver carcinogen, causes less degranulation than 3-aminopyrene. Similarly, the test is not specific for liver carcinogens, compounds as diverse in action as some polycyclic hydrocarbons and 2-naphthylamine also giving positive results.

Some caution is needed when this work is compared with that of other laboratories. It is well known that microsomal activity against a variety of substrates shows wide variation between rats of different strains (Page and Vesell, 1969) and age (Shoemaker and Hamrick,

1974). Differing conditions of husbandry, and the nutritional state of the animals also have effects on the cytochrome P450 levels and subsequently on the enzymic activity of microsomes (Vesell, 1967; Vesell *et al.*, 1972; Basu and Dickerson, 1974). All these factors could, therefore, influence the metabolism of carcinogens.

Figure IV.1 illustrates a dose-response curve for 3,3′-DCB. Similar curves were obtained using other carcinogens. The data reflect the care needed in choosing dose levels in degranulation assays. Increasing the carcinogen concentration above a certain level decreases the degranulation. The concentrations used in the evaluation screen were those falling in the area of maximum degranulation.

### REFERENCES

Basu, T. K. & Dickerson, J. W. T. (1974) Interrelationships of Nutrition and the Metabolism of Drugs. *Chem. Biol. Interact.*, **8**, 193.

Borton, P., Carson, R. & Reed, D. J. (1974) Stability of Rat Liver Microsomes Quick Frozen with Liquid Nitrogen and Stored at − 85°C. *Biochem. Pharmacol.*, **23**, 2332.

Flaks, B. (1970) Changes in the Fine Structure of Rat Hepatocytes during the Early Phases of Chronic 2-acetylaminofluorene Intoxication. *Chem. Biol. Interact.*, **2**, 129.

Fleck, A. & Begg, D. (1954) The Estimation of Ribonucleic Acid Using Ultraviolet Absorption Measurements. *Biochem. biophys. Acta.*, **108**, 333.

Fleischer, S. & Kervina, M. (1974) Subcellular Fractionation of Rat Liver. *Meth. Enzym.*, **31** (Part A), 6.

Garner, R. C. (1973) Chemical Evidence for the Formation of a Reactive Aflatoxin $B_1$ Metabolite by Hamster Liver Microsomes. *FEBS Letters*, **36**, 261.

Gaunce, A. P. & D'Iorio, A. (1970) Microdetermination of Protein by an Automated Lowry Method. *Anal. Biochem.*, **37**, 204.

Ketterer, B., Holt, S. J. & Ross-Mansell, P. (1967) The Effect of a Single Intraperitoneal Dose of the Hepatocarcinogen 4-dimethylaminoazobenzene on the Rough Surfaced Endoplasmic Reticulum of the Liver of the Rat. *Biochem. J.*, **103**, 692.

Loeb, J. M., Howell, R. R. & Tomkins, G. M. (1965) Turnover of Ribosomal RNA in Rat Liver. *Science*, **149**, 1093.

Lowry, O. H., Rosebrough, N. J., Farr, A. L. & Randall, R. J. (1951) Protein Measurement with the Folin Phenol Reagent. *J. biol. Chem.*, **193**, 265.

Page, J. G. & Vesell, E. S. (1969) Hepatic Drug Metabolism in Ten Strains of Norway Rat Before and After Pretreatment with Phenobarbital. *Proc. Soc. exp. Biol. Med.*, **131**, 256

Porter, K. R. & Bruni, C. (1959) An Electron Microscope Study of the Early Effects of 3′-Me-DAB on Rat Liver Cells. *Cancer Res.*, **19**, 997.

Purchase, I. F. H. & Lefevre, P. A. (1975) Rapid Tests for Carcinogens. *Chemy. Indy.*, **10**, 415.

Sabatini, D. D., Tashiro, Y. & Palade, G. E. (1966) On the Attachment of Ribosomes to Microsomal Membranes. *J. mol. Biol.*, **19**, 503.

Shoemaker, D. D. & Hamrick, M. E. (1974) Stoichiometry of Drug Metabolism in Maturing Male Rats. *Biochem. Pharmacol.*, **23**, 2325.

Svoboda, D. & Higginson, J. (1968) A Comparison of Ultrastructural Changes in Rat Liver due to Chemical Carcinogens. *Cancer Res.*, **28**, 1703.

Swenson, D. H., Miller, J. A. & Miller, E. C. (1973) 2,3-Dihydro-2,3-dihydroxyaflatoxin $B_1$: an Acid Hydrolysis Product of an RNA Aflatoxin $B_1$ Adduct Formed by Hamster and Rat Liver Microsomes. *Biochem. biophys. Res. Commun.*, **53**, 1260.

Vesell, E. S. (1967) Induction of Drug Metabolising Enzymes in Liver Microsomes of Mice and Rats by Softwood Bedding. *Science*, **157**, 1057.

Vessell, E. S., Lang, C. M., White, W. J., Passananti, G. T. & Tripp, S. L. (1972) Hepatic Drug Metabolism in Rats: Impairment in a Dirty Environment. *Science*, 179, 896.

Williams, D. J. & Rabin, B. R. (1969) Disruption by Carcinogens of the Hormone Dependent Association of Membranes with Polysomes. *Nature*, **232**, 102.

Williams, D. J. & Rabin, B. R. (1969) The Effects of Aflatoxin $B_1$ and Steroid Hormones on Polysome Binding to Microsomal Membranes as Measured by the Activity of an Enzyme Catalysing Disulphide Interchange. *FEBS Letters*, **4**, 103.